# An ultra-low-power image compressor for capsule endoscope

**DOI:** 10.1186/1475-925X-5-14

**Published:** 2006-02-25

**Authors:** Meng-Chun Lin, Lan-Rong Dung, Ping-Kuo Weng

**Affiliations:** 1 Department of Electrical and Control Engineering National Chiao Tung University, Hsinchu, Taiwan; 2 Solid-State Devices Section, Materials and Electro-Optics Research Division, Chung-Shan Institute of Science and Technology, Lung-Tan, Tao-Yuan, Taiwan

## Abstract

**Background:**

Gastrointestinal (GI) endoscopy has been popularly applied for the diagnosis of diseases of the alimentary canal including Crohn's Disease, Celiac disease and other malabsorption disorders, benign and malignant tumors of the small intestine, vascular disorders and medication related small bowel injury. The wireless capsule endoscope has been successfully utilized to diagnose diseases of the small intestine and alleviate the discomfort and pain of patients. However, the resolution of demosaicked image is still low, and some interesting spots may be unintentionally omitted. Especially, the images will be severely distorted when physicians zoom images in for detailed diagnosis. Increasing resolution may cause significant power consumption in RF transmitter; hence, image compression is necessary for saving the power dissipation of RF transmitter. To overcome this drawback, we have been developing a new capsule endoscope, called GICam.

**Methods:**

We developed an ultra-low-power image compression processor for capsule endoscope or swallowable imaging capsules. In applications of capsule endoscopy, it is imperative to consider battery life/performance trade-offs. Applying state-of-the-art video compression techniques may significantly reduce the image bit rate by their high compression ratio, but they all require intensive computation and consume much battery power. There are many fast compression algorithms for reducing computation load; however, they may result in distortion of the original image, which is not good for use in the medical care. Thus, this paper will first simplify traditional video compression algorithms and propose a scalable compression architecture.

**Conclusion:**

As the result, the developed video compressor only costs 31 K gates at 2 frames per second, consumes 14.92 mW, and reduces the video size by 75% at least.

## Background

Gastrointestinal (GI) endoscopy has been popularly applied for the diagnosis of diseases of the alimentary canal including Crohn's Disease, Celiac disease and other malabsorption disorders, benign and malignant tumors of the small intestine, vascular disorders and medication related small bowel injury. There exist two classes of GI endoscopy; wired active endoscopy and wireless passive capsule endoscopy. The wired active endoscopy can enable efficient diagnosis based on real images and biopsy samples; however, it causes patients discomfort and pain to push flexible, relatively bulky cables into the digestive tube. To relief the suffering of patients, wireless passive capsule endoscopes are being developed worldwide [1-4]. The capsule moves passively through the internal GI tract with the aid of peristalsis and transmits images of the intestine wirelessly.

**Figure 1:**
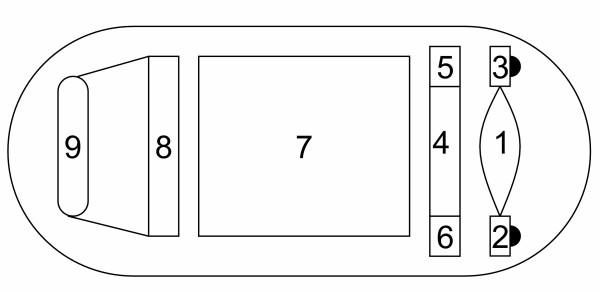
The system structure of GICam (1: Len; 2,3: LEDs; 4: CMOS sensor; 5: Image compressor; 6: Scanline controller; 7: Battery; 8: RF transmitter; 9: Antenna).

The state-of-the-art is the commercial wireless capsule endoscope product, the PillCam capsule, developed by Given Imaging Ltd. The PillCam capsule transmits the GI images at the resolution of 256-by-256 8-bit pixels and the frame rate of 2 frames/sec (or fps). The PillCam has been successfully utilized to diagnose diseases of the small intestine and alleviate the discomfort and pain of patients. However, based on clinical experience; the PillCam still has some drawbacks. First, the PillCam cannot control its heading and moving direction itself. This drawback may cause image oversights and miss a disease. Second, the resolution of demosaicked image is still low, and some interesting spots may be unintentionally omitted. Especially, the images will be severely distorted when physicians zoom images in for detailed diagnosis. The first drawback is the nature of passive endoscopy. Some papers have presented approaches for the autonomous moving function [5,6]. Very few papers address the solutions of the second drawback. Increasing resolution may alleviate the second problem; however, it would result in significant power consumption in RF transmitter. Hence, applying image compression is necessary for saving the power dissipation of RF transmitter. The paper [11] provides a thorough review on GI image compression and motivated our research. To overcome the second drawback, we have been developing a new capsule endoscope, called GICam. Fig. 1 illustrates the system diagram of the proposed capsule endoscope. We attached an ultra-low-power image compressor to the CMOS sensor to deliver a compressed 512-by-512 image while the RF transmission rate is at 2 megabits per second. To reduce the buffer size between the CMOS sensor and the image compressor, the scanline controller is dedicated to scan out R, G1, G2, and B signals in a certain order.

The scope of this paper is the design of an image compression processor for capsule endoscopes. Instead of applying existing compression standards, we developed simplified image compression specifically for capsule endoscopes. Unlike the general image compression techniques, the proposed image compression starts from raw images in the format of Bayer patterns and processes R, G1, G2, and B signals separately. Comparing with the traditional image compression, the proposed image compression is low-powered for three reasons. First, the proposed image compression does not need demosaicking, and hence saves the computing power of interpolation steps. Second, the proposed compression starts from the raw image, and does not need inner product operations for color-space transformation. Finally, the computation load of the 8-by-8 discrete cosine transform (DCT) can be reduced by the factor of 3.

## Methods

### The proposed image compression algorithm

Traditional image compression algorithms use the optimized quantization for YC_b_C_r _image to reduce compressed image size while the visual distortion is low. In order to quantize YC_b_C_r _image, the typical image compression requires two preprocessing steps that are demosaicking and the color space transformation. However, the demosaicking step requires weighted sums for color interpolation and the color space transformation requires calculation of inner products. From the view point of GICam, it is not worth it to dissipate power for both preprocessing steps as long as the compression quality and ratio are acceptable. The measure of compression quality is the peak signal-to-noise ratio (*PSNR*). The calculation of *PSNR *is formulated as Eq. (1):



Where *MSE *is the mean square error of decompressed image. The compression ratio (*CR*) is defined as the ratio of the raw image size to the compressed image size. The measure of the compression ratio is the compression rate. The formula of the compression rate is calculated by Eq. (2):

*compression rate *= (1-*CR*^-1^) × 100%     (2)

**Figure 2:**
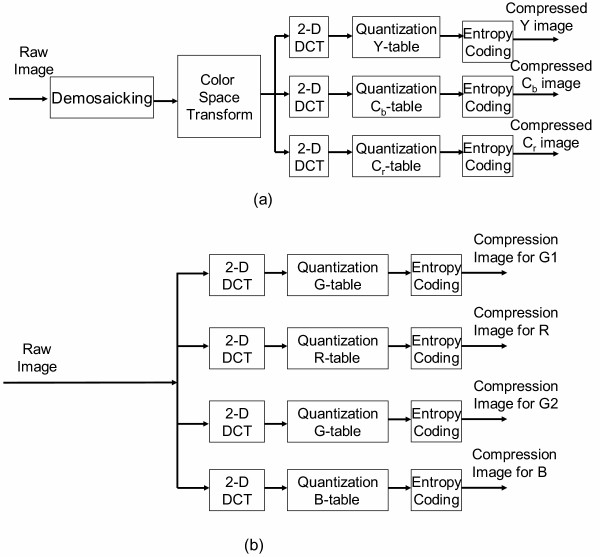
(a) A typical image compression algorithm (b) The GICam image compression algorithm.

**Figure 3:**
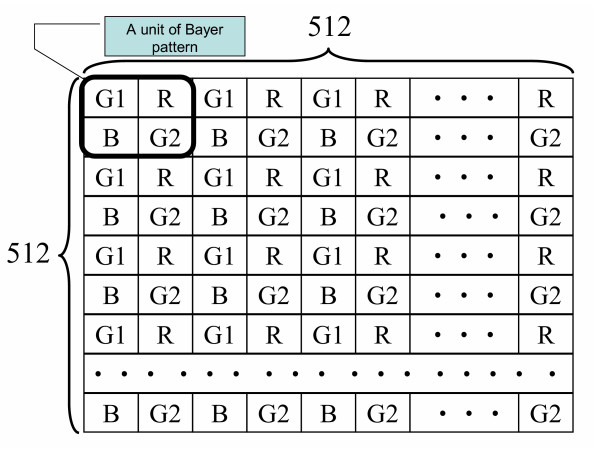
The Bayer patterns in the raw image.

**Figure 4:**
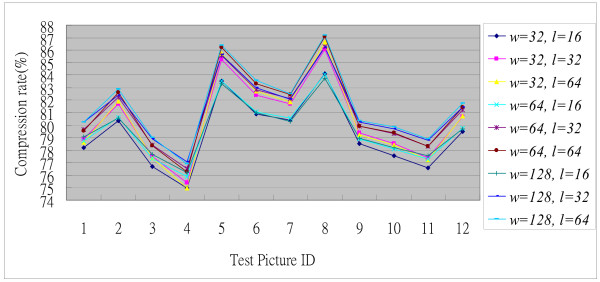
The modified RGB quantization table.

Fig. 2 illustrates the power saving on the proposed image compression. First of all, the GICam image compression directly processes raw images without demosaicking and color space transform. For a 512 × 512 image, when using the Bayer format, the image has 256 × 256 Bayer patterns. Fig. 3 shows the Bayer patterns in the CMOS image sensor. So, the incoming image size to the 2D-DCT is 256 × 256 × 8 × 4 bits, where each pixel is an 8-bit datum and each of R, G1, G2, and B components has 256 × 256 pixels. Since the image size after preprocessing in the traditional algorithm is 512 × 512 × 8 × 3 bits, the computational load of 2D-DCT and quantization is reduced by the factor of 3. Traditional compression algorithms employ the YC_b_C_r _quantization to earn a good compression ratio while the visual distortion is minimized, based on the factors related to the sensitivity of the human visual system (HVS). However, for the sake of power saving, our compression rather uses the RGB quantization to save the computation of demosaicking and color space transformation. According to [7], the RGB quantization can result in similar decompressed image quality as the YC_b_C_r _quantization. As mentioned above, the advantage of applying RGB quantization is two-fold: saving the power dissipation on preprocessing steps and reducing the computing load of 2D-DCT and quantization. Although the RGB quantization for the Bayer-formatted image requires four quantizing products, the number of tables is three in that G1 and G2 components can share the same *green *quantization table. Moreover, to reduce the hardware cost and quantization power dissipation, we modified the RGB quantization tables in [7] as shown in Fig. 4. In the modified tables, the quantization multipliers are power-of-two's. As shown in the simulation result, the degradation of compressed image is low when comparing with the original RGB quantization. The minor shortcoming of the RGB quantization is that the quantization latency is longer than the YC_b_C_r _quantization when the R-G1-G2-B quantizations are pipelined. Thanks to the low frame rate specification in capsule endoscopy, the increasing of quantization latency is acceptable.

In GICam, the Lempel-Ziv (LZ) coding [8] is employed for the entropy coding. The reason why we adopted the LZ coding as the entropy coding is that the LZ encoding does not need look-up tables and complex computation. Thus, the LZ encoding can consume less power and use smaller silicon size than the other candidates, such as the Huffman encoding and the arithmetic coding.

**Figure 5:**
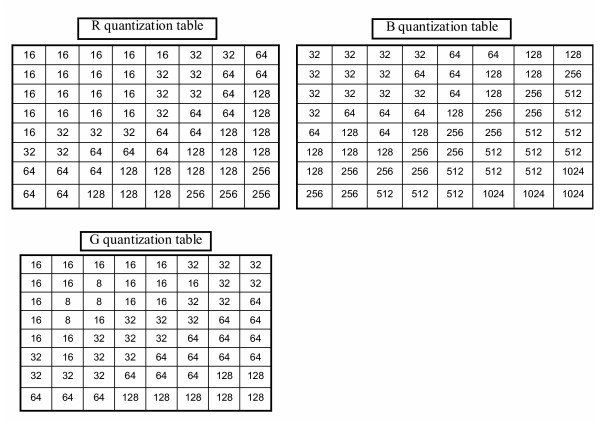
The simulation results of the GICam image compression.

**Figure 6:**
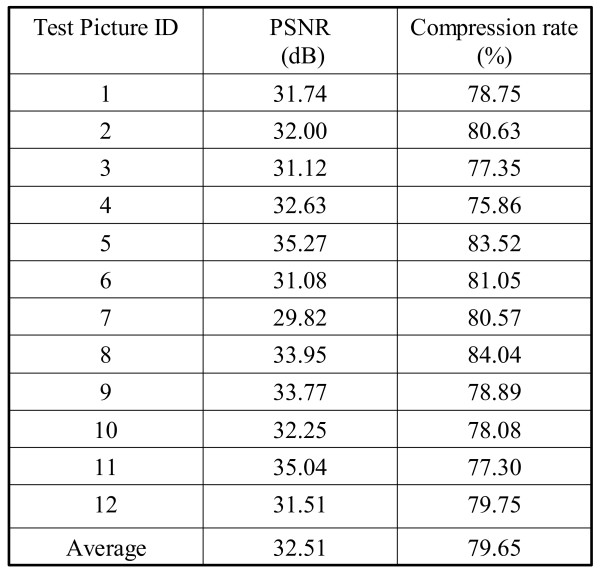
The simulation results of twelve tested pictures.

**Figure 7:**
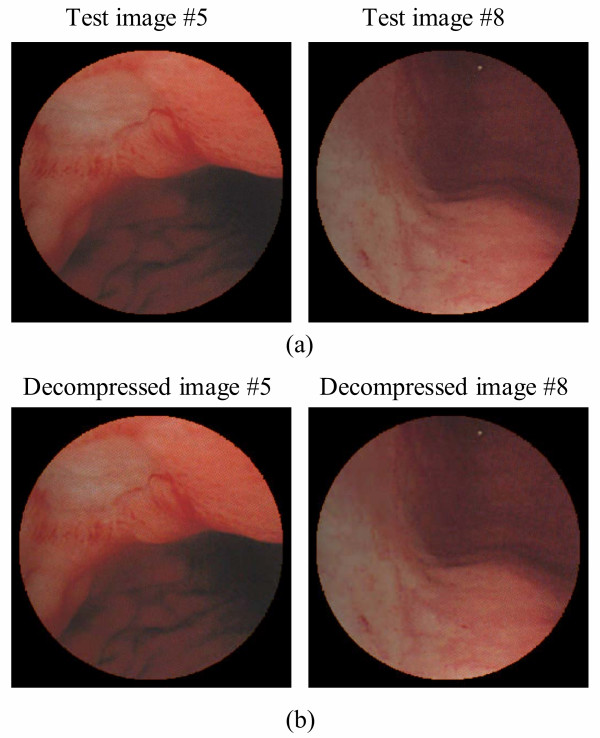
(a) Demosaicked images from raw images #5 and #8. (b) Demosaicked images from decompressed images #5 and #8.

The target compression performance of the GICam image compression is to reduce image size by 75% at least. To meet the specification, given the quantization tables, we exploited the cost-optimal LZ coding parameters. There are two parameters in the LZ coding to be determined; they are the window size, *w*, and the maximum matching length, *l*. The larger the parameters, the higher the compression ratio but the higher the implementation cost. As per the experimental results shown in the Fig. 5, the increase in compression ratio becomes very slow, as the parameters are large; however, the implementation cost keeps growing linearly. Hence, we set the values of parameters by using the compression ratio of 4:1 as the threshold. Our goal is to determine the minimum (*w*, *l*) set under the constraint of 4:1 compression ratio. The results in Fig. 5 are collected by simulating the candidate LZ encoding schemes with the 8-by-8 2D-DCT and the RGB quantization. As seen in Fig. 5, simulating with 12 endoscopic pictures, (64, 16) is the minimum (*w*, *l*) set to meet the compression ratio requirement. Using (64, 16) as the parameter set, in Fig. 6, we can see the performance in terms of the quality degradation and compression ratio. The result shows that the degradation of decompressed images is quite low while the average PSNR is 32.51 dB. The original image involved in the PSNR calculation is the Bayer pattern image. According to the objective criterion of medical doctors the PSNR higher than 30 dB is acceptable. To demonstrate the results, Fig. 7 illustrates the compression quality of two test pictures. The difference between the original image and the decompressed image is invisible.

### Architecture design and implementation of GIcam image compressor

**Figure 8:**
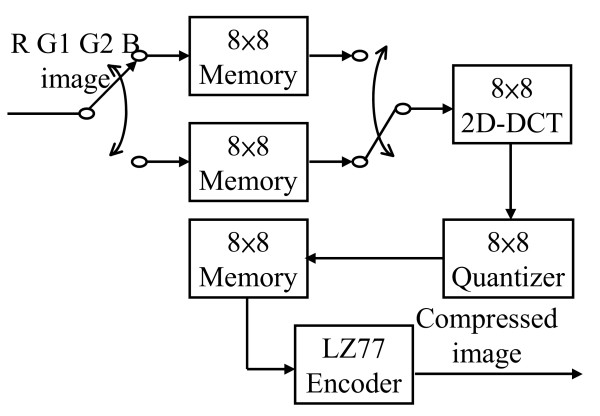
The architecture of GICam image processor.

Fig. 8 shows the architecture of the GICam image compressor. The GICam image compressor processes the image in the order of R, G1, G2 and B. Because the data stream from the image sensor is block-based, the GICam image compressor requires intermediate memory units to hold each block of data. Because the 2D-DCT is a row-column recursive structure, its input data are queued by a set of ping-pong buffers. In addition, the 8-by-8-memory array between the quantizer and the LZ77 encoder is used to synchronize the operations of quantization and LZ77 encoding. Since the frame rate of GICam is 2 frames/second, the 2D-DCT can be folded to trade the hardware cost with the computing speed, and the other two data processing units, quantization and LZ77 encoder, can operate at low data rate.

**Figure 9:**
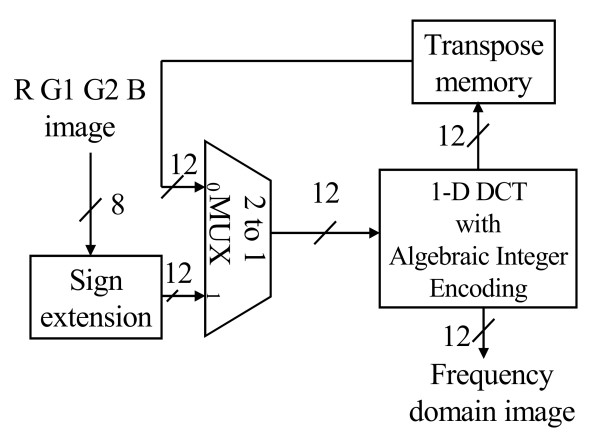
The block diagram of 2D-DCT.

**Figure 10:**
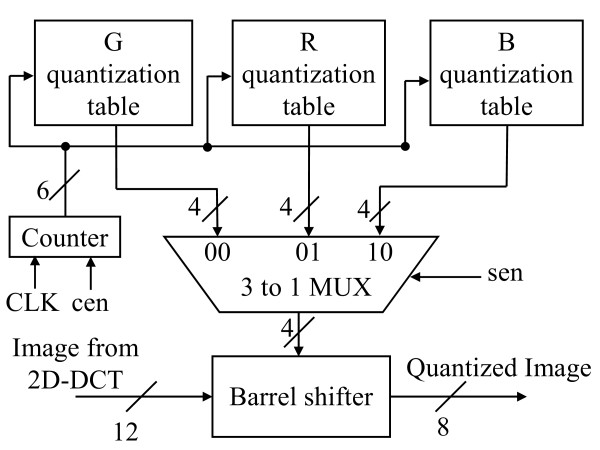
The block diagram of Quantizer.

**Figure 11:**
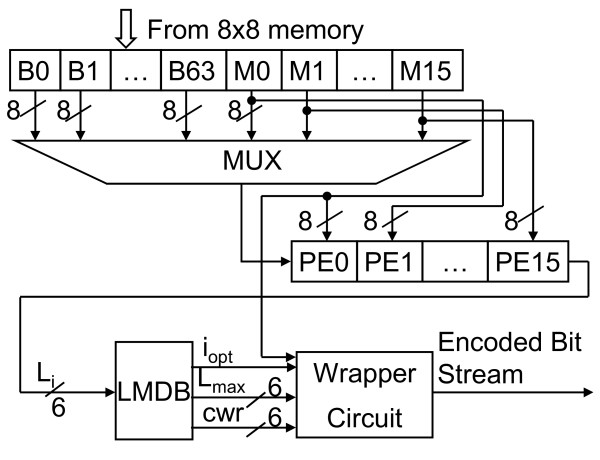
The block diagram of LZ 77 encoder. (LMDB: Longest match length decision block)

**Figure 12:**
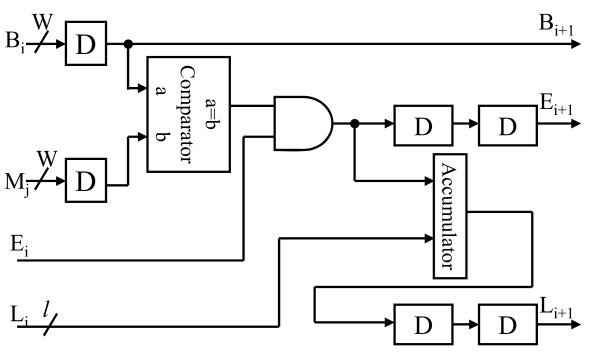
The circuit of PE in the LZ77 encoder.

Fig. 9 illustrates the block diagram of 2D-DCT. The 2D-DCT alternatively calculates row or column 1D-DCTs. The 1D-DCT is a multiplier-less implementation using the algebraic integer encoding [10]. The algebraic integer encoding can minimize the number of addition operations. Doing so, we can produce a low-cost, power saving DCT datapath. According to the report from PrimePower™, the logic part of 2D-DCT consumes 1.53 mW@1.57 MHz and the transpose memory costs 2.80 mW. As regards the RGB quantizer, the GICam image processor utilizes the barrel shifter for power-of-two products. The power-of-two quantization table shown in Fig. 4 can reduce the cost of multiplication while quality degradation is quite little. As shown in Fig. 10, we use the barrel shifter to perform the quantization. According to the PrimePower™ report, the quantization consumes 0.115 mW. Finally, the LZ77 encoder is implemented by block-matching approach as shown in Fig. 11[9]. The detail of each processing element (PE) is shown in Fig. 12. As the result of simulation, the power consumption of LZ77 is 3.87 mW.

**Figure 13:**
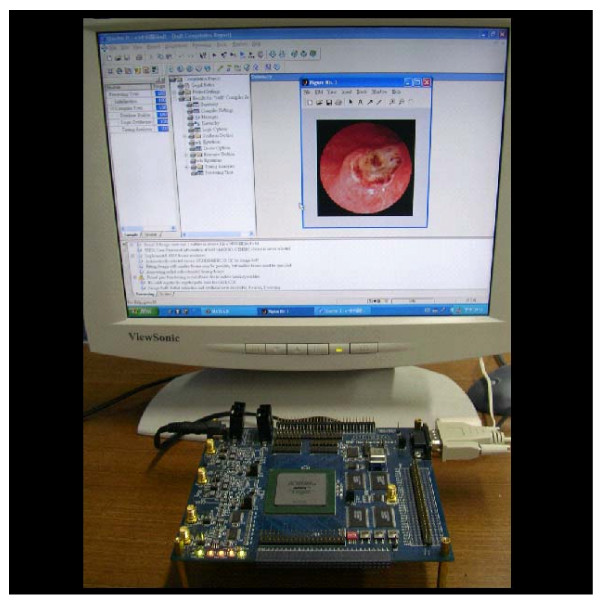
The FPGA prototype of the CICam image compressor.

**Figure 14:**
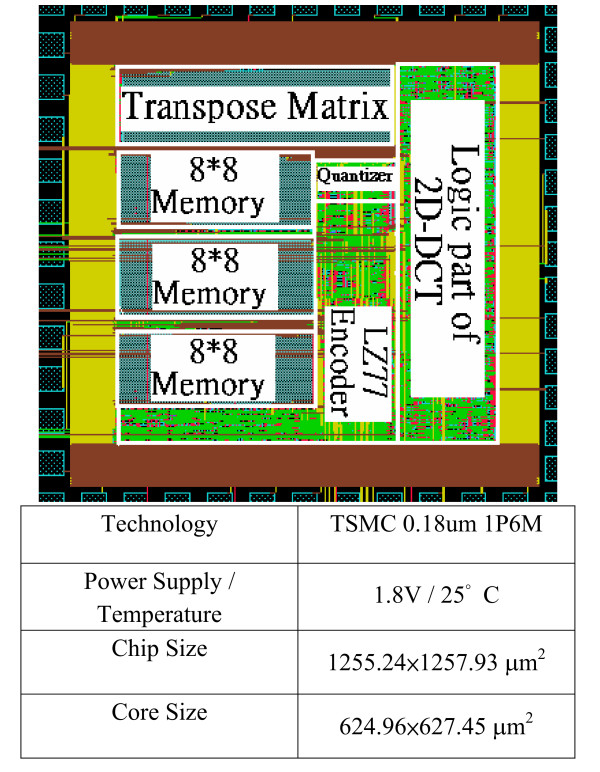
The layout of the GICam image compressor.

**Figure 15:**
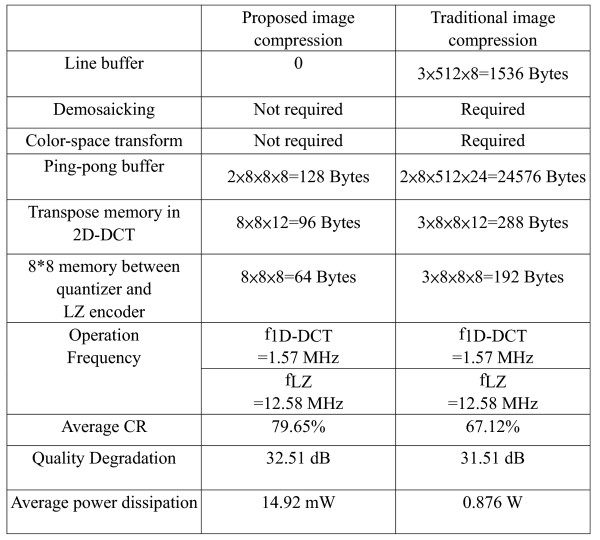
The comparison of proposed image compression and the traditional image compression applying for GICam application.

To validate the GICam image processor, we used the FPGA board of Altera APEX2100 K to verify the function of the GICAM image processor and the prototype is shown in Fig. 13. Test results are the same as simulation results of the algorithm level using MTALAB. After FPGA verification, we used the TSMC 0.18 μm 1P6M process to implement the GICam image compressor. The logic part is synthesized by using Synopsys Design Analyzer™. The gate count of 2D-DCT, quantizer, and LZ77 encoder is 31 K gates. There are two clocks in the chip. One at 1.57 MHz is for 2D-DCT and Quantizer, and another at 12.58 MHz is for LZ77 encoder. When operating at 1.8 V, the power consumption of logic part is 5.52 mW, estimated by using PrimePower™. The memory blocks are generated by Artisan memory compiler and consume 9.40 mW. Fig. 14 illustrates the layout of the GICam image compressor. When comparing the proposed image compression with the traditional one in Fig. 15, the power dissipation can save 98.2% because of the reduction of memory requirement. Except comparing with the traditional one, we further analysis the power saving from system perspective. For a 512-by-512 GI images, if we do not use the proposed image compressor to compress the data of GI image, the total power dissipation is 33.5 mW, in which, the sensor consumes 8 mW, the RF transmitter consumes 24 mW and LEDS consumes 1.5 mW respectively. However, the GICam compresses the GI image and total dissipation power is 33.5 mW. The power dissipation of the RF transmitter can be reduced to 6 mW and the proposed image compressor consumes 14.92 mW. Hence, using the proposed image compressor can efficiently save the total power dissipation of 3.08 mW and substantially reduce the damage of the human body health from the RF transmitter.

## Conclusion

This paper presents an ultra-low-power image compression processor for capsule endoscope or swallowable imaging capsules. In applications of capsule endoscopy, it is imperative to consider battery life/performance trade-offs. Instead of applying state-of-the-art video compression techniques, we propose an RGB-based compression algorithm in which the memory size and computational load can be significantly reduced. We first simplified traditional video compression algorithms by removing the color-space transformation. As shown in the result, the developed video compressor only costs 31 K gates at 2 frames per second, consumes 14.92 mW, and reduces the video size by 75% at least.
